# Effectiveness of adding a workplace intervention to an inpatient multimodal occupational rehabilitation program: A randomized clinical trial

**DOI:** 10.5271/sjweh.3873

**Published:** 2020-07-01

**Authors:** Martin Skagseth, Marius S Fimland, Marit B Rise, Roar Johnsen, Petter C Borchgrevink, Lene Aasdahl

**Affiliations:** Department of Public Health and Nursing, Faculty of Medicine and Health Sciences, Norwegian University of Science and Technology, Trondheim, Norway; Hysnes Rehabilitation Center, St. Olavs Hospital, Trondheim, Norway; Unicare Helsefort Rehabilitation Centre, Rissa, Norway; Department of Neuromedicine and Movement Science, Faculty of Medicine and Health Sciences, Norwegian University of Science and Technology, Trondheim, Norway; Department of Mental Health, Faculty of Medicine and Health Sciences, Norwegian University of Science and Technology, Trondheim, Norway; Department of Circulation and Medical Imaging, Faculty of Medicine, Norwegian University of Science and Technology, Trondheim, Norway; National Competence Centre for Complex Disorders, St. Olavs University Hospital, Trondheim, Norway

**Keywords:** Key terms RCT, return to work, RTW, sickness absence, sick leave

## Abstract

**Objectives:**

This study aimed to evaluate the effectiveness of a workplace intervention (WI) added to an inpatient multimodal occupational rehabilitation program (I-MORE) on sickness absence.

**Methods:**

In this researcher-blinded randomized controlled trial with parallel groups, individuals on sick leave due to musculoskeletal, unspecified- or common mental health disorders were randomized to I-MORE (N=87) or I-MORE+WI (N=88). I-MORE lasted 2+1 weeks (with one week at home in between) and consisted of “acceptance and commitment therapy”, physical exercise, and work-related problem solving. The additional WI consisted of a preparatory part, a workplace meeting involving the sick-listed worker, the employer, and the primary rehabilitation therapist at the rehabilitation center, and follow-up work related to the meeting. The primary outcomes were number of sickness absence days and time until sustainable return to work (RTW) during 12 months of follow-up, measured by registry data.

**Results:**

The median number of sickness absence days during the 12-month follow-up for I-MORE was 115 days [interquartile range (IQR) 53–183] versus 130 days (IQR 81–212) for I-MORE+WI. The difference between groups was not statistically significant (P=0.084). The hazard ratio for sustainable RTW was 0.74 (95% confidence interval 0.48–1.16; P=0.192) in favor of I-MORE.

**Conclusions:**

This study provided no evidence in favor of I-MORE+WI compared to only I-MORE for long-term sickness absent individuals with musculoskeletal-, common mental- or unspecified disorders.

Sickness absence is a vast challenge in the western world, with consequences both for the sick-listed worker and society (1–3). Thus, several return-to-work (RTW) interventions have been assessed in the past decades with inconsistent results (4–9). However, studies suggest that multimodal interventions (10) could be important, particularly when adding workplace interventions (WI) (10–13). Most of these studies have recruited sick-listed workers with musculoskeletal complaints and low-back pain. Although studies also suggest that WI could be promising for individuals with common mental health disorders (6), the results are inconsistent (13).

In Norway, 3–4 weeks of inpatient multimodal occupational rehabilitation (I-MORE) is common for long-term sick-listed individuals with complex biopsychosocial barriers for RTW. Such programs are usually transdiagnostic, consisting of physical exercise, cognitive behavioral therapy, patient education, and work-related problem solving (14–16). In a recent randomized clinical trial, we found that a 3.5-week I-MORE reduced sickness absence compared with an outpatient cognitive behavioral intervention (unpublished data) (17, 18). Still, very few of the participants had been in contact with the workplace or their employer during the rehabilitation program (19). Thus, we adjusted the 3.5-week program to include a WI. This randomized clinical trial compared I-MORE to I-MORE+WI. We hypothesized that adding WI to I-MORE would lead to faster sustainable RTW and less sickness absence days.

## Methods

This researcher-blinded randomized clinical trial (RCT)comparing parallel groups is described in detail in the protocol article (20). The Regional Committee for Medical and Health Research Ethics in Central Norway approved the study (No:2014/2279), which was registered at clinicaltrials.gov (NCT02541890). The results are presented according to the CONSORT statement (21).

### Participants

Individuals living in Trøndelag county in Central Norway were eligible to participate if they: (i) were aged 18–60 years, (ii) were sick listed 2–12 months, (iii) were employed in at least a 20% position (eg, ≥1 day per week), (iv) had an employer, (v) had a sick leave status of ≥50% off work, (vi) anticipated ≥4 more weeks of sick-leave, and (vii) had a diagnosis within the musculoskeletal, psychological or general and unspecified chapters of International Classification Primary Care, version 2 (ICPC-2). Potential participants were recruited in one of two ways: identified in registers from the Norwegian Labor and Welfare Administration (NAV) and invited through a letter or referred from their general practitioner. At the outpatient screening clinic, a physician, psychologist, and a physiotherapist assessed eligibility. Exclusion criteria were any of the following: being self-employed, having or being under consideration for a serious somatic or mental health/substance abuse disorder, currently undergoing rehabilitation, having significant problems with working in a group, insufficient comprehension of Norwegian language to participate in group sessions and to complete questionnaires, scheduled for surgery within the next six months, or being pregnant.

### Interventions

*I-MORE*. The I-MORE program took place at Hysnes Rehabilitation Center, established as a part of St. Olavs Hospital in Trøndelag, Norway. The program lasted four weeks: two weeks at the rehabilitation center, one week at home, and one week at the center. The program consisted mainly of “acceptance and commitment therapy” (ACT; third generation cognitive behavioral therapy) (22), physical exercise training, and group- and individual sessions of work-related problem-solving resulting in a RTW plan. See table 1 for more information about the full content. An interdisciplinary team consisting of a psychologist, physiotherapist, exercise physiologist, nurse, physician and welfare caseworker provided the rehabilitation program. A certified ACT instructor supervised the coordinators monthly during the intervention. In addition to the multidisciplinary team, each participant was appointed a primary rehabilitation therapist as a contact person. The participants had several individual meetings with their primary rehabilitation therapist designing the RTW plan. The experiences from the week at home were used to try out new coping strategies and adjust the RTW plan. A more detailed description of the program has been published elsewhere (20).

**Table 1:** Components in the two programs [I-MORE=inpatient multimodal occupational rehabilitation; WI=workplace intervention]

Content	I-MORE	I-MORE+WI
Acceptance and commitment therapy (8 group sessions)	x	x
Physical activity (group sessions and individual guidance)	x	x
Lectures (stress, sleep, nutrition, pain)	x	x
Mindfulness sessions (group based)	x	x
Work-related problem solving with primary ­rehabilitation therapist	x	x
Individual return to work plan	x	x
Group meeting with workplace-related topics (1 session)		x
Individual preparation to workplace meeting		x
Workplace meeting (employer, employee, coordinator)		x
Summary from the workplace meeting		x

*I-MORE+WI*. The workplace intervention consisted of (i) preparations before the workplace meeting, (ii) the workplace meeting, and (iii) writing a summary of the meeting (table 1). The preparations consisted of using a part of the scheduled meetings between the participant and their primary rehabilitation therapist to discuss the workplace meeting. In addition, there was a group meeting to talk about expectations, challenges and the value of RTW. The coordinator contacted the participant`s employer before the meeting. They informed the employer about the agenda for the meeting, which included using a booklet called *A Conversation about Work Possibilities*. Developed by NAV, the booklet is a function assessment tool and often used for RTW problem solving (23). It contains questions about the individual`s work and potential barriers for RTW and is a tool developed for professionals working with sick-listed individuals. The workplace meeting took place in week three (the week at home). The aim of the meeting was to discuss possibilities and progress for RTW. The meeting was scheduled for two hours and most commonly included the participant, the employer, and the primary rehabilitation therapist. The participant’s general physician and/or labor and welfare caseworker at NAV were informed about the meeting and was involved when appropriate. The rehabilitation therapist contacted the employer after the meeting to ensure that the plans and actions agreed upon in the meeting was followed up and completed. The participant and the rehabilitation therapist discussed the outcome and experiences from the meeting. A summary from the meeting, concluding what had been agreed upon, was written and sent to all participants. The summary was also added to the RTW plan and sent to the general practitioner and the NAV case worker.

### Study context

In Norway, all legal residents are included in the public insurance system. Medically certified sick leave is compensated 100% the first 12 months. The employer pays for the first 16 days and the remainder is paid by NAV. It is encouraged to consider graded sick leave (20–100%), independent of employment fraction. After 12 months of sick leave, it is possible to apply for more long-term medical benefits: work assessments allowance and permanent disability benefits, both of which compensate approximately 66% of prior income.

### Outcome measures

During 12 months of follow-up, register data was collected for sick leave payments, sick leave certificates, work assessment allowance and disability pension. The primary outcome measures were (i) cumulated number of sickness absence days during 12 months of follow-up after inclusion and (ii) time until sustainable RTW in the follow-up period, defined as four weeks without receiving medical benefits.

### Randomization

Eligible individuals who passed the outpatient screening were randomized to I-MORE or I-MORE+WI. Block randomization with unknown sizes was performed by a web-based program delivered by a third party, the Unit for Applied Clinical Research at the Norwegian University of Science and Technology. It was not possible to blind the participants and the caregivers. Researchers were blinded until the analyses were completed.

### Sample size

To analyze between-group differences with Kaplan Meier survival analysis with the log rank test with a hazard ratio (HR) of 0.6 (alpha 0.05, beta 0.20), would require 63 participants in each group. The use of register-based sickness absence data eliminates loss to follow-up in the intention to treat analysis of primary outcomes. To provide enough statistical power for questionnaire-based outcomes, the aim was 80 participants in each group (20). The sample size estimation was based on results in the field (24–26).

### Statistical analysis

Sickness absence was registered both as number of days per month and as a dichotomous measure of whether or not the participant was registered on sick leave that month. We used monthly intervals (rather than exact dates) in order to contain all relevant sick leave benefits in the same measure, as exact dates were not available for payments and the long-term benefits. Number of days on sick leave was recalculated from a seven- to five-day work-week. Time on graded sick leave was recalculated to whole sick leave days. Sick leave days were adjusted for employment fraction and any increase in disability pension during follow-up was counted as sick leave.

Number of sickness absence days during 12 months follow-up after inclusion for the two groups were compared using the Mann-Whitney U (Wilcoxon rank sum) test, as sick leave days were not normally distributed. To analyze time to sustainable RTW, Kaplan Meier curves were estimated and compared with the log rank test. To estimate HR for RTW, we used the Cox proportional hazard model and the Efron method for ties (27). Time was calculated as the number of months until RTW, and participants were censored at full sustainable RTW or end of follow-up. Analyses were performed without adjustments and adjusted for age, gender, education, main diagnosis and length of sick leave at inclusion. The proportionality hazard assumption was checked using the Schoenfeld Residual test (28). The main analyses were performed in line with the intention-to-treat principle. In addition, we performed per protocol analyses excluding participants that withdrew after randomization (before or during the program) or did not want to do the workplace visit.

All analyses were done using STATA 14 (StataCorp, College Station, TX, USA).

## Results

**Figure 1:**
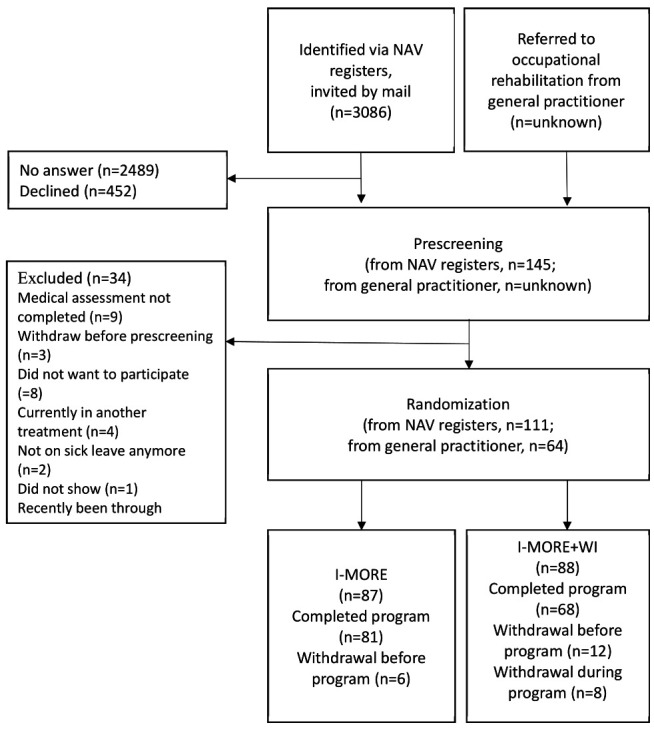
Participation flow through the study. NAV registers=social security register from Norwegian Labor and Welfare Administration; I-MORE=inpatient multimodal occupational rehabilitation; I-MORE+WI=inpatient multimodal occupational rehabilitation with workplace intervention.

The flow of participants in the study is presented in figure 1. In total, 3086 potential participants were identified in the registers from NAV (between January 2015 and June 2016) and invited to take part in the study. Of these, 145 accepted the invitation and were invited for an outpatient screening at St Olavs Hospital. The number of patients referred from general practitioners to the outpatient screening was not available. After the outpatient screening, 175 participants were included in the study (111 from NAV registers and 64 referred from their general practitioner). All the workplace meetings in the WI were executed (100%), and lasted approximately two hours, as scheduled. Main themes in the meetings were the participants’ health and work situation, the RTW process, perceived barriers during this process, as well as potential solutions.

### Participants’ characteristics

The participants were mainly women (79%) and the mean age was 46 (SD 9) years. About half had higher education (55%), and most worked full time (71%) prior to their sick leave, while 28% worked part time, and one individual had a graded disability pension. The median number of sickness absence days during the 12 months prior to inclusion was 184 days [interquartile range (IQR) 139–255]. A sick leave diagnosis within the musculoskeletal (44%) and psychological (43%) chapters of ICPC-2 were most common, while 13% were diagnosed with a general and unspecific diagnosis (chapter A). The baseline characteristics of the participants in the two programs were fairly similar (table 2).

**Table 2:** Baseline characteristics of the participants. [HADS=hospital anxiety and depression scale; I-MORE=inpatient multimodal occupational rehabilitation; I-MORE+WI=inpatient multimodal occupational rehabilitation with workplace intervention; IQR=interquartile range; SD=standard deviation]

	I-MORE+WI (N=88)						I-MORE (N=87)					
	
	Mean	SD	N	%	Median	IQR	Mean	SD	N	%	Median	IQR
Age	45	9					46	8				
Women			68	77					70	80		
Higher education ^a^			42	52					49	85		
Diagnosis												
A-general and unspecified			13	15					9	11		
L-musculoskeletal			36	43					39	46		
P-psychological			35	42					37	44		
HADS												
Anxiety (0–21)	8.4	4.5					7.6	4.4				
Depression (0–21)	6.6	4.2					6.8	4.2				
Length of sick leave at inclusion ^b^					184	137–242					184	144–268
Average pain level (0–10)	6.9	5.2					6.9	4.2				
Work status before sick leave												
Full job			61	70					63	73		
Part-time			26	30					22	26		
Partly on disability pension	0								1	1		

a College or university

b Number of days on sick leave during the last 12 months prior to inclusion. Measured as calendar days, not adjusted for partial sick- leave. Based on data from the national social security system registry.

### Sickness absence

**Figure 2:**
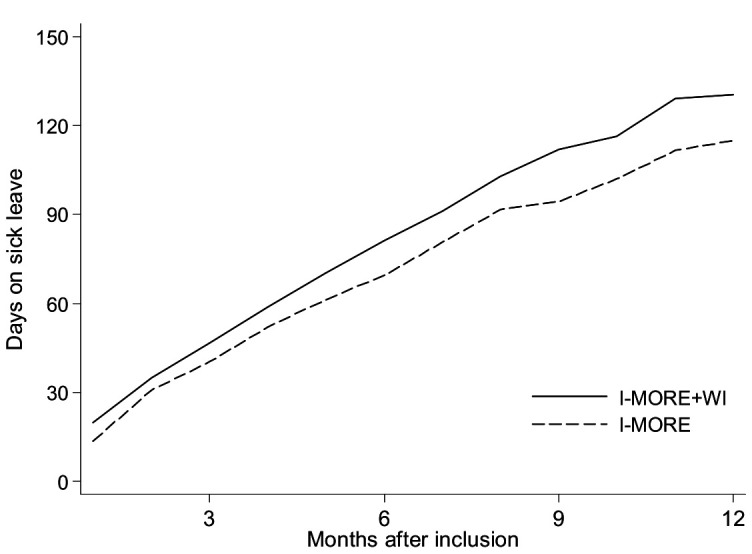
Cumulative number of days (median) on medical benefits for participants in standard inpatient multimodal occupational rehabilitation (I-MORE; dashed line) and standard inpatient multimodal occupational rehabilitation with workplace intervention (I-MORE+WI; solid line) during 12 months of follow-up. Adjusted for employment fraction and transformed to whole workdays according to a 5-day work week.

During 12 months of follow-up, the median number of sickness absence days for I-MORE+WI was 130 days (IQR 81–212), and 115 days (IQR 53–183) for I-MORE (figure 2). The difference between the groups was not statistically significant (Mann-Whitney U test, P=0.084).

**Figure 3:**
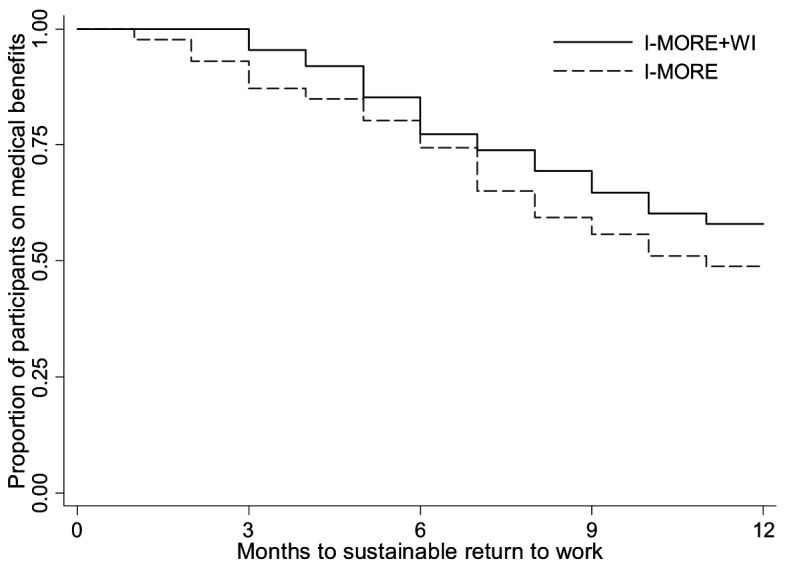
Survival curves from the Kaplan Meier analysis showing time to sustainable return to work for participants in standard inpatient multimodal occupational rehabilitation (I-MORE; dashed line) and standard inpatient multimodal occupational rehabilitation with workplace intervention (I-MORE+WI; solid line) during 12 months of follow-up.

In total, 46.8% (82 participants) achieved sustainable RTW during 12 months of follow-up; 37 participants (42%) in I-MORE+WI and 45 participants (52%) in I-MORE. The Kaplan Meier plot is shown in figure 3. The difference between the programs was not statistically significant (log rank test: P=0.190). The Cox regression analysis without adjustment gave a HR of 0.74 [95% confidence interval (CI) 0.48–1.16, P=0.192], in favor of I-MORE. The adjusted analysis showed similar results (HR 0.77, 95% CI 0.49–1.23, P=0.286). The per-protocol analysis did not provide any substantial changes in the estimates (results not shown).

## Discussion

In contrast to our hypothesis, WI added to I-MORE did not facilitate work participation among adults on long-term sick leave with musculoskeletal, common mental or unspecified disorders. Rather, the estimates indicate that participants in I-MORE+WI versus I-MORE had an unfavorable impact from the WI, although there were no statistically significant differences between the programs.

Our results contrast with systematic reviews which conclude that WI in multimodal occupational rehabilitation increases RTW (10, 11). The results are also in contrast to comparable randomized clinical trials on WI and RTW (25, 26, 29, 30). Loisel et al (26) found that patients with low-back pain receiving a combined clinical and workplace intervention returned to regular work 2.4 times faster than those receiving usual care and that the workplace intervention accounted for most of the effect. The model was later replicated in the Netherlands where Anema et al (29) reported a RTW HR of 1.7 for the workplace intervention group compared to the control group. In another study, Bültmann et al (31) found 30% less sickness absence hours for an intervention including a workplace intervention compared to a more limited intervention. However, it should be noted that most of the aforementioned studies included participants with considerably shorter sick leave duration than our study (26, 29, 30); all were aimed at musculoskeletal disorders (25, 26, 29, 30) and none was conducted in an inpatient setting. However, despite including participants on long-term sick leave (median 150 days), Lambeek et al (25) found a substantial effect of integrated care for workers with chronic low-back pain (88 days versus 208 days until RTW) compared to the usual care group. Still, the intervention was quite extensive, in contrast to our study where the only difference between the groups was the WI.

Coordination between stakeholders is considered a key factor in helping sick-listed workers return to work (10, 11, 13, 32–35). In their systematic review, Carrol et al (11) concluded that involving the workplace alone is insufficient and that coordination between stakeholders is necessary to increase RTW. The lack of coordination between stakeholders in the present study could therefore explain that there was no effect of the WI. Moreover, a previous study from our research group showed that I-MORE was effective in reducing sickness absence (unpublished data) (17, 18), the room for additional improvement from WI might be limited. Furthermore, the I-MORE program also included a work focus with work related problem solving and making of an RTW-plan, which might have made the difference between the groups too small to add effect from the I-MORE. Even though there were no statistically significant differences between the programs, the estimates indicate more sickness absence days and a delayed RTW for I-MORE+WI. Although this should be interpreted with caution, it is possible that the WI somehow interfered with the RTW process.

The study was performed in a Norwegian context where the employer only pays for the 16 first days of sick leave, after which national insurance pays the remaining period of sick leave. Hence, there is not clear economic incentive for the employer to help the employee return to work. However, all the employers in this study agreed to participate in the workplace intervention, indicating a wish to take part in the employee`s RTW process. Sickness absence is reimbursed 100% of the salary for the first 12 months in Norway. It could be argued that this takes away the incentive to return to work as fast as possible. However, the median sick leave before inclusion in the current study was 184 days, ie, during follow-up most participants would expend their sick leave period (maximum one year) and would then have to apply for more long-term benefits, which are only reimbursed 66%.

The main strength of this randomized study was the use of registry data for sickness absence measurements, ensuring no recall bias and no missing data. A limitation is the lack of information about the number of participants referred from general practitioners, as information was only available for the number of individuals that passed the outpatient screening. In addition, of the 3086 invitation letters that were sent out, only 145 (5%) accepted, limiting the generalizability of the results. Furthermore, potential participants had to be willing to do a workplace meeting, hence individuals with a problematic relationship with their employer or workplace, may have declined to participate in the study. In RCT, it is recommended to perform analyses adjusted for important predictors. A limitation in this study is the lack of information about participants’ expectations at the start of the program. However, we performed sensitivity analyses adjusted for other important predictors such as education and length of sick leave at inclusion. Blinding of the participants or the healthcare providers at the rehabilitation center was not possible, however, the researchers were blinded, and two researchers performed the analyses separately.

### Concluding remarks

When added to the I-MORE, the current WI did not facilitate work participation among individuals on long-term sick leave with musculoskeletal, common mental or unspecified disorders. Hence, this study provides no evidence in support of supplementing I-MORE with this limited WI for the current disorders. More research is needed on how the workplace can be effectively included in occupational rehabilitation.

### Competing interest

The authors declare no conflicts of interest.

### Funding

The Norwegian Government allocated funding through the Central Norway Regional Health Authority and St. Olavs Hospital, Trondheim, and the Research Council of Norway.
